# Physical-model guided self-distillation network for single image dehazing

**DOI:** 10.3389/fnbot.2022.1036465

**Published:** 2022-12-01

**Authors:** Yunwei Lan, Zhigao Cui, Yanzhao Su, Nian Wang, Aihua Li, Deshuai Han

**Affiliations:** Xi'an Research Institute of High Technology, Xi'an, China

**Keywords:** image dehazing, knowledge distillation, attention mechanism, deep learning, computer vision

## Abstract

**Motivation:**

Image dehazing, as a key prerequisite of high-level computer vision tasks, has gained extensive attention in recent years. Traditional model-based methods acquire dehazed images *via* the atmospheric scattering model, which dehazed favorably but often causes artifacts due to the error of parameter estimation. By contrast, recent model-free methods directly restore dehazed images by building an end-to-end network, which achieves better color fidelity. To improve the dehazing effect, we combine the complementary merits of these two categories and propose a physical-model guided self-distillation network for single image dehazing named PMGSDN.

**Proposed method:**

First, we propose a novel attention guided feature extraction block (AGFEB) and build a deep feature extraction network by it. Second, we propose three early-exit branches and embed the dark channel prior information to the network to merge the merits of model-based methods and model-free methods, and then we adopt self-distillation to transfer the features from the deeper layers (perform as teacher) to shallow early-exit branches (perform as student) to improve the dehazing effect.

**Results:**

For I-HAZE and O-HAZE datasets, better than the other methods, the proposed method achieves the best values of PSNR and SSIM being 17.41dB, 0.813, 18.48dB, and 0.802. Moreover, for real-world images, the proposed method also obtains high quality dehazed results.

**Conclusion:**

Experimental results on both synthetic and real-world images demonstrate that the proposed PMGSDN can effectively dehaze images, resulting in dehazed results with clear textures and good color fidelity.

## Introduction

Images captured under haze condition have abnormal brightness and low contrast, which affects the further application in high-level computer vision tasks, such as image super-resolution (Chen et al., 2021a,b) and semantic segmentation. Hence, image dehazing, as a key prerequisite of high-level computer vision tasks, becomes a significant subject in recent years. Generally, the formation of hazy images can be modeled as Equation 1, atmospheric scattering model (also called as physical-model):


(1) $$\begin{array}{l} I\left(x\right)=J\left(x\right)t\left(x\right)+A\left(1-t\left(x\right)\right) \end{array}$$

where *I* represents images obtained under haze condition; *J* represents haze-free images; *x* represents the pixel location; *A* and *t* represent the atmospheric light and transmission map, respectively. Obviously, we cannot directly restore the haze-free images *J* from the given hazy images *I* since both the atmospheric light *A* and transmission map *t* are undetermined.

To address this problem, early methods use priors obtained from the statistical rule on haze-free images to estimate the atmospheric light and transmission map, then dehaze images *via* the atmospheric scattering model, including dark channel prior (DCP) (He et al., 2011), color-lines prior (CLP) (Fattal, 2014), color attenuation prior (CAP) (Zhu et al., 2015), and non-local dehazing (NLD) (Berman et al., 2016). These methods dehaze favorably in special scenes but tend to over enhance images since unilateral assumptions cannot fit in all situations. With the development of deep learning, some methods (Cai et al., 2016; Ren et al., 2016; Li et al., 2017; Zhang and Patel, 2018) adopt convolutional neural network (CNN) to estimate the atmospheric light and transmission map more accurately and obtain better dehazed images based on the atmospheric scattering model. However, the atmospheric scattering model is an ideal equation, which cannot sufficiently represent the formation of hazy images. Hence, these methods still cause some halos and color distortions.

To solve the problem, some end-to-end dehazing networks (Chen et al., 2019; Liu X. et al., 2019; Qu et al., 2019; Dong et al., 2020; Qin et al., 2020; Zhao et al., 2020) are proposed, which directly restore dehazed images by establishing the mapping between hazy and haze-free images instead of using the atmospheric scattering model. These model-free methods can produce dehazed images with better color fidelity. However, due to trained on synthetic datasets, these model-free methods can perform well on synthetic images but always acquire under-dehazed results when applied to real scenes since synthetic images cannot represent uneven haze distribution and complex illumination condition existing in real scenes. To this end, some novel end-to-end methods (Hong et al., 2020; Shao et al., 2020; Chen et al., 2021; Zhao et al., 2021) combine with model-based methods and achieve better dehazing effects in real scenes. However, these methods cannot exploit features from different depths to improve the guidance efficiency of extra knowledge.

**Figure 1:**
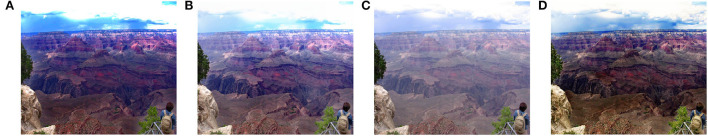
Comparative results on a real-world image. **(A)** High contrast result with some color distortion generated by DCP. **(B)** High contrast result with some illumination distortion generated by DCPDN. **(C)** Under-dehazed result with better color fidelity generated by MSBDN. **(D)** Our result, which combines their merits.

According to the above analyses, we summarize that the existing model-based dehazing methods can effectively restore image texture details but tend to cause color changes and artifacts. By contrast, model-free dehazing methods directly obtain dehazed images with good color fidelity by supervised training. But the dehazing effect is often limited in natural scenes since the training samples are synthetic images. Thus, to improve the dehazing effect, we merge the merits of these two categories *via* self-distillation and propose a physical-model guided self-distillation network for single image dehazing. Moreover, we compare the dehazing effect of the above algorithms on a real-world image. The experimental results are shown in Figure 1. The model-based methods [DCP (He et al., 2011) and DCPDN (Zhang and Patel, 2018)] can restore dehazed images with discriminative textures but suffer from some color and illumination overenhancement. The model-free method MSBDN (Dong et al., 2020) can maintain color fidelity but acquire an under-dehazed image. Better than the other methods, the proposed PMGSDN combines the complementary merits of model-free methods and model-based methods, and obtains high quality dehazed results with natural color and rich details.

**Figure 2:**
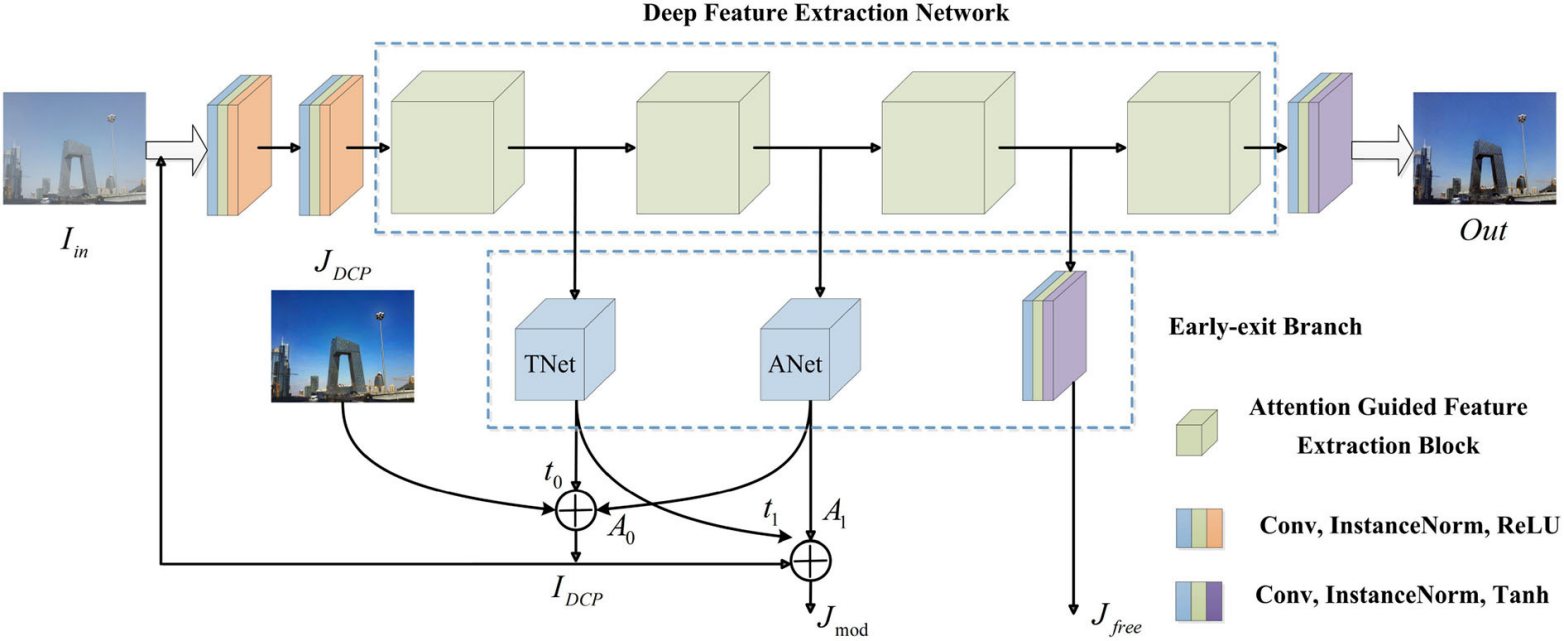
The general network structure of PMGSDN. TNet, the network adopted to estimate transmission map. ANet, the network adopted to estimate atmospheric light.

As shown in Figure 2, we first build a deep feature extraction network (DFEN) constructed with four attention guided feature extraction blocks (AGFEBs) to effectively extract features from different depths. Moreover, we add three early-exit branches to acquire intermediate dehazed images and optimize the network by a two-stage training strategy. In the first stage, we obtain the preliminary transmission map *t*_0_ and atmospheric light *A*_0_ by two early-exit branches and embed dark channel prior (DCP) into the network to acquire the preliminarily dehazed images *J*_*DCP*_ base on the hazy input *I*_*in*_. Hence, reconstructed hazy images *I*_*DCP*_ can be obtained by substituting the *J*_*DCP*_, *A*_0_, and *t*_0_ into the atmospheric scattering model. In the second stage, we feed the *I*_*DCP*_ into the network and obtain the final dehazed images *Out*, the intermediate model-free dehazed images *J*_*free*_, and model-based dehazed images *J*_mod_ (produced by substituting the intermediate transmission map *t*_1_, atmospheric light *A*_1_, and the *I*_*DCP*_ into the model). Considering that these intermediate dehazed images have complementary advantages in terms of image contrast and color fidelity, we combine the merits of them by a one-stage knowledge distillation (see **Figure 4**), which transfers the knowledge from deeper layers (performs as a teacher) to shallow layers (performs as a student) within the network. We call this distillation strategy as self-distillation, which achieves the joint training and optimization of both teacher and students. For this article, the main contributions are as follows:

1. To improve the dehazing effect, we merge the merits of both model-based dehazing methods and model-free dehazing methods, and propose a physical-model guided self-distillation network for single image dehazing named PMGSDN.
2. In order to improve the feature extraction ability of the network for different depths, we propose an attention guided feature extraction blocks (AGFEB) to construct the deep feature extraction network.
3. To reduce the dependence of the student network on the pretrained teacher model and improve the efficiency of knowledge distillation, we propose a self-distillation strategy and embed the dark channel prior information to the network to further improve the dehazing effect.

## Related work

### Model-based methods

Model-based methods restore haze-free images using the atmospheric scattering model, where the estimation of transmission map and atmospheric light is a key prerequisite. Early model-based methods (also called prior-based methods) explore various priors concluded from the statistic rule to estimate transmission map and atmospheric light, and then dehaze images *via* the atmospheric scattering model. For example, the dark channel prior (DCP) (He et al., 2011) estimate transmission map based on the observation that clear images have low intensity in at least one of the RGB channels. The color-lines prior (CLP) (Fattal, 2014) constructs a model based on the color lines and estimates the transmission map using the lines' offset. Differently, the color attenuation prior (CAP) (Zhu et al., 2015) builds a linear model to estimate the scene depth and transmission map based on the difference between the brightness and saturation of hazy images. Another method no-local dehazing (NLD) (Berman et al., 2016) estimates the transmission map and acquires dehazed images *via* the hundreds of distinct colors. These prior-based methods achieve favorable dehazing effects but suffer from severe distortion and artifacts.

Recently, some methods estimate transmission map and atmospheric light more accurately by data driving and acquire dehazed images with fewer artifacts. For instance, Ren et al. propose a multi-scale convolution neural network (MSCNN) (Ren et al., 2016) to estimate the transmission map in a coarse-to-fine way. Another method DehazeNet (Cai et al., 2016) adopts Maxout units to effectively extract features and estimate the transmission map. Differently, AODNet (Li et al., 2017) combines these two parameters into one parameter to restore dehazed images. DCPDN (Zhang and Patel, 2018) embeds the atmospheric scattering model into CNN to estimate the atmospheric light and transmission map. These two methods estimate the transmission map and atmospheric light simultaneously and alleviate the cumulative error of two parameter estimations. However, due to the atmospheric scattering model being a simplified model, which cannot sufficiently represent the formation of hazy images, the above two model-based methods still suffer from color and illumination changes.

### Model-free methods

Model-free methods directly restore dehazed images *via* an end-to-end network without using the atmospheric scattering model. Due to a huge gap between the features of hazy images and haze-free images, these methods usually increase the network scales and depths to enhance feature extraction ability. For example, the MSBDN (Dong et al., 2020) constructs a multi-scale boosting dehazing network to combine the features from different scales by a dense feature fusion module. FFA (Qin et al., 2020) effectively extracts features and restores dehazed images using a deep network constructed with feature attention blocks. Moreover, GridDehazeNet (Liu X. et al., 2019) and GCANet (Chen et al., 2019), respectively adopt attention mechanisms and gated fusion networks to effectively fuse multi-scale features. Differently, the EPDN (Qu et al., 2019) builds a generative adversarial network to improve the dehazing effect by the adversarial learning between a multi-scale generator and discriminator. Another dehazing method (Zhao et al., 2020) adopts the cycle generative adversarial network to alleviate the paired training constraint. These methods perform well on synthetic images but tend to fail to deal with real-world images due to being trained on synthetic datasets. To address this problem, DA (Shao et al., 2020) builds a bidirectional network to reduce the gap between real-word and synthetic images. PSD (Chen et al., 2021) adopts a committee consists of multi priors to guide the network training and acquire high contrast images but suffer from illumination changes, and RefineDNet (Zhao et al., 2021) embeds DCP and the atmospheric scattering model to reconstruct hazy images and then improves the model's generalization ability *via* unpaired adversarial training. Moreover, some methods also improve deep learning-based algorithms in other computer vision tasks by introducing additional knowledge. For example, Xia et al. (2022) improved the Kernel Correlation Filter algorithm to address the problem that the object tracking algorithm fails to track under the influence of occlusion conditions. Chen et al. (2021c) proposed an image completion algorithm based on an improved total variation minimization method.

### Knowledge distillation

Knowledge distillation is first proposed by Hinton (Hinton et al., 2015) to compress the model by transferring the knowledge from a cumbersome teacher network to a compact student network. Recently, knowledge distillation is also applied to the model enhancement through improved learning strategy [including self-learning (Ji et al., 2021; Zheng and Peng, 2022) and mutual learning (Li et al., 2021)]. For example, Hong et al. (2020) applies knowledge distillation to heterogeneous task imitation and guides the student network training using the features extracted from the image reconstruction task. Liu Y. et al. (2019) adopts structure knowledge distillation to transfer the knowledge from a large network to a small semantic segmentation network since semantic segmentation is a structured prediction problem. These two distillation methods both start with a powerful but cumbersome teacher network (a pretrained network) and perform one-way knowledge transfer to a compact student network (a network to be trained). However, two shortcomings exist in them: a powerful teacher network is not always available; a two-stage training process is not efficient. Hence, online distillation and self-distillation are proposed to implement the joint training and optimization of both teacher and student (one-stage training process) by improved learning strategies. Typically, Li et al. (2021) builds a multi-branch network and acquires predicted heatmaps from each branch, which are then assembled (performs as a teacher) to teach each branch (performs as a student) in reverse. However, this method simply aggregates students to form an assembled teacher, which restrains the diversity of students and cannot exploit features from different depths of the network. Hence, we applied self-distillation (Zhang et al., 2021) into our PMGSDN to enhance the generalization ability in real scenes.

## Proposed method

### Overall structure

As shown in Figure 2, the PMGSDN contains three parts: preprocessing model, a deep feature extraction network, and early-exit branches. In the preprocessing model, we first adopt two 3 × 3 convolutions to preprocess the hazy input *F*_*in*_ and change its shape to 32 × 256 × 256, where each convolution is followed by an instance normalization and ReLU function. Moreover, these two convolutions have different parameter settings, the input channel, output channel, kernel size, stride, and padding of the first convolution are 3, 32, 3, 1, and 1, respectively, and the corresponding parameters of the second convolution are set to 32, 32, 3, 1, and 1.

#### Deep feature extraction network

To effectively extract features from different depths, we feed the preprocessed features into the deep feature extraction network (DFEN) constructed with four attention guided feature extraction blocks (AGFEBs). After that, a convolution followed by an instance normalization and the Tanh function is utilized to produce the final dehazed images *Out*. The parameter settings of the convolution used here are set to 32, 3, 3, 1, and 1, respectively.

**Figure 3:**
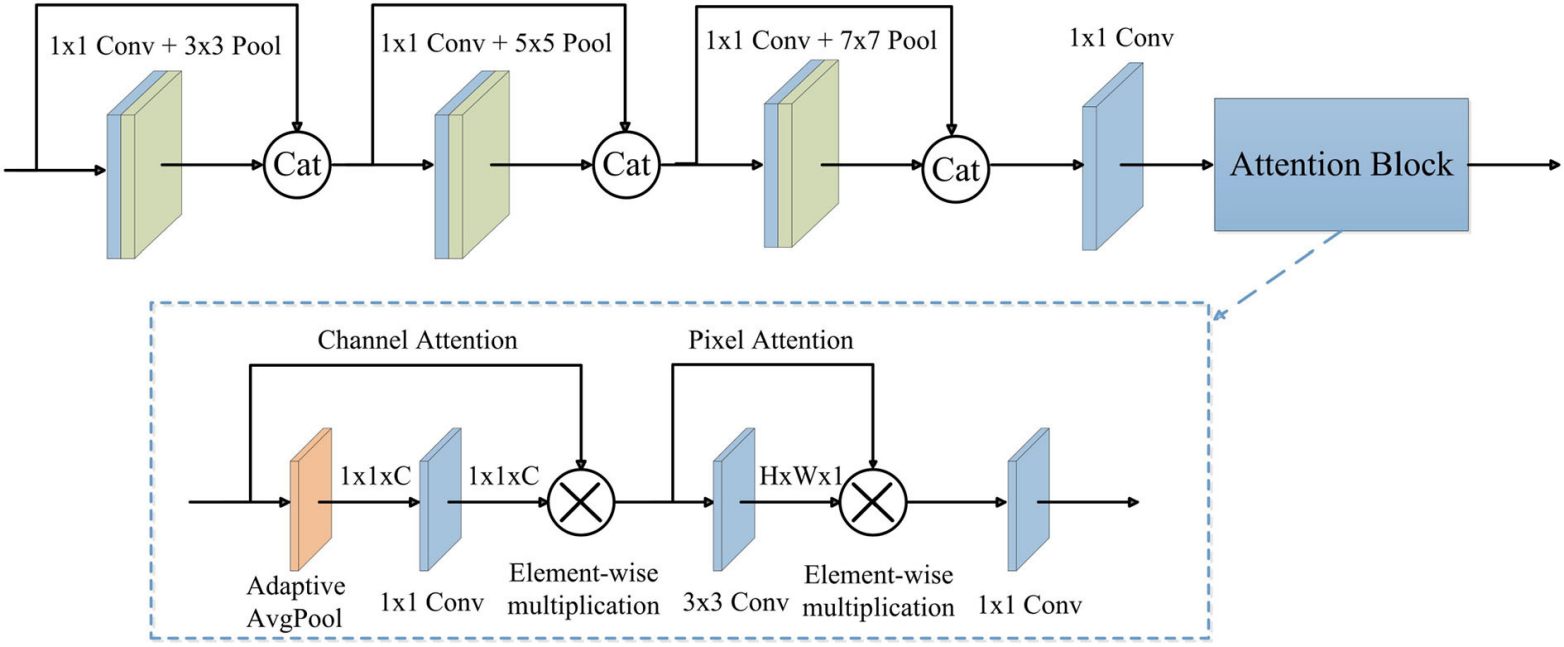
The structure of AGFEB. Cat, channel-wise concatenation.

As shown in Figure 3, the proposed AGFEB first extracts features using four convolutions. These convolutions are all point-wise convolutions (1 × 1 convolution) (Zhang and Tao, 2020), where the first three convolutions with pooling layers form different receptive fields and the fourth convolution is utilized for dimension reduction. Note that we replace traditional convolutions with the kernel size of 3 × 3, 5 × 5, and 7 × 7 to point-wise convolutions with 3 × 3, 5 × 5, and 7 × 7 pooling layer, and thus the AGFEB is computationally efficient since no large convolutional kernel is used. Moreover, the first three convolutions combine the features of the current convolution with the features of the last one by channel-wise concatenation to obtain more abundant features. After that, we introduce an attention block consisting of channel attention, pixel attention, and a point-wise convolution to make the network pay more attention to improve feature representation. During the channel attention, an adaptive average pooling is firstly used to generate a channel vector with the shape of 1 × 1 × C and then a 1 × 1 convolution followed by a sigmoid function is utilized to produce channel attention maps, which are used to weigh these inputs *via* element-wise multiplication. After the channel attention, the enhanced features can concern different channel maps unequally and effectively alleviate the global color distortions. Different from the channel attention, the pixel attention first adopts a 3 × 3 convolution followed by a sigmoid function to generate spatial attention maps and then weights the input by element-wise multiplication to pay more attention to high frequency regions, such as textures and structures. Finally, we adopt the point-wise convolution to change the shape to 32 × 256 × 256 and get the output. The parameter settings of the proposed AGFEB are shown in Table 1.

**Table 1:** The parameter settings of the proposed AGFEB.

	**Conv1**	**Pool1**	**Conv2**	**Pool2**	**Conv3**	**Pool3**	**Conv4**	**Adaptive Avgpool**	**Conv5**	**Conv6**	**Conv7**
Input channel	32	–	64	–	96	–	128	–	32	32	32
Output channel	32	–	32	–	32	–	32	–	32	1	32
Kernel size	1	3	1	5	1	7	1	1	1	3	1
Stride	1	1	1	1	1	1	1	–	1	1	1
Padding	0	1	0	2	0	3	0	–	0	1	0

The convolution and pooling used in AGFEB are expressed as Conv 1 to Conv 7 and Pool 1 to Pool 3 from left to right and top to bottom. Notice that the kernel size of the adaptive average pooling represents the target output size of the feature.

#### Early-exit branches

To combine both model-based methods and model-free methods, we add three early-exit branches after each AGFEB. The first two branches are named as TNet and ANet to estimate the transmission map and atmospheric light respectively and then acquire the intermediate dehazed images by the atmospheric scattering model. The details of the TNet and ANet can be seen in article (Zhang and Patel, 2018). Moreover, the third branch is constructed with a convolution, an instance normalization, and the Tanh function, which directly acquires intermediate dehazed images in a model-free way, and the parameter settings of the convolution used here are set to 32, 3, 3, 1, and 1, respectively.

### Forward prediction and self-distillation

To effectively combine the complementary merits of model-based methods and model-free dehazing methods, we divide the training process into two parts: forward prediction and self-distillation.

#### Forward prediction

As shown in Figure 2, we divide the forward prediction into two stages. In the first stage, we send the input hazy images *I*_*in*_ into the PMGSDN, and obtain the preliminary transmission map *t*_0_ and atmospheric light *A*_0_ by the first two early-exit branches. Meanwhile, we embed dark channel prior (DCP) (He et al., 2011) into a network to acquire the preliminary dehazed images *J*_*DCP*_. Hence, based on the atmospheric scattering model, reconstructed hazy images *I*_*DCP*_ can be produced, which can be expressed as Equation 2:


(2) $$\begin{array}{l} I_{DCP}=J_{DCP}t_{0}+A_{0}\left(1-t_{0}\right) \end{array}$$

Compared with the synthetic hazy images *F*_*in*_, the reconstructed hazy images *I*_*DCP*_ are more similar to real-world hazy images since the DCP is a statistical rule based on the observation of haze-free images. Hence, in the second stage, we regard the reconstructed hazy images *I*_*DCP*_ as the input of PMGSDN and acquire the final dehazed images *Out* by the deep feature extraction network (DFEN). Similar to the first stage, the intermediate transmission map *t*_1_ and atmospheric light *A*_1_ are generated to acquire the model-based dehazed images *J*_mod_. Differently, the model-free dehazed images *J*_*free*_ are generated simultaneously by the third early-exit branch.

#### Self-distillation

**Figure 4:**
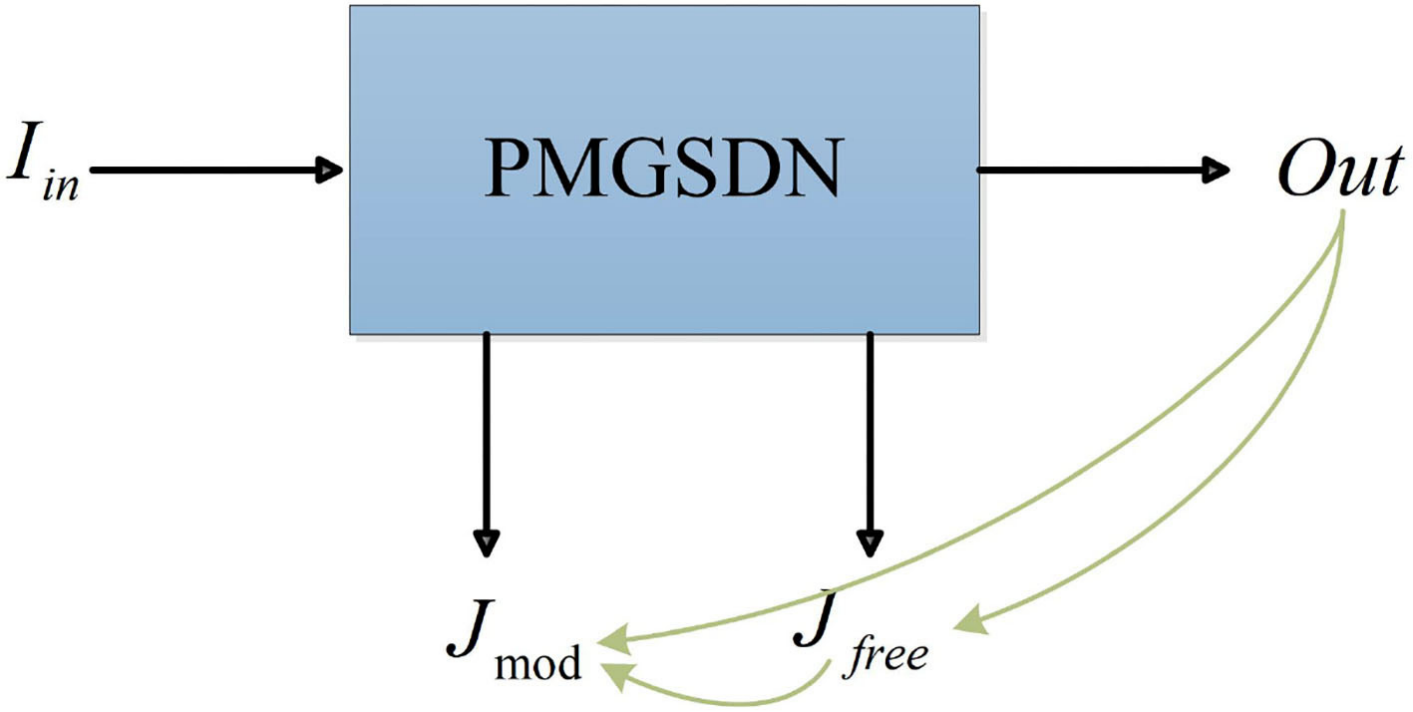
Self-distillation.

The intermediate dehazed images *J*_mod_ and *J*_*free*_ are generated by the features from different depths and have complementary advantages in terms of image contrast and color fidelity in local regions. Hence, we adopt a one-stage knowledge distillation called self-distillation to effectively combine the merits of them. As shown in Figure 4, we propose a self-distillation strategy, which builds extra distillation loss among intermediate model-based dehazed images *J*_mod_, model-free dehazed images *J*_*free*_, and the final dehazed images *Out*. In this way, the final dehazed images *Out* combine the features from different depths and improve the generalization ability of a model.

### Loss function

Several experiments (Liu et al., 2020; Fu et al., 2021) have proven that the combination of pixel-wise and feature-wise loss can effectively improve training efficiency. Hence, the overall loss consists of reconstruct loss and distillation loss, which can be expressed as Equation 3:


(3) $$\begin{array}{l} L_{loss}=L_{rec}+L_{dist} \end{array}$$

where *L*_*loss*_ represents the overall loss, *L*_*rec*_ represents the reconstruct loss, and *L*_*dist*_ represents the distillation loss.

#### Reconstruct loss

Previous work (Qin et al., 2020) has shown that pixel-wise loss can rapidly match the feature distribution between the dehazed images and ground truths. Different from L2 loss (mean square error), L1 loss (standard deviation error) can make the network training more stable. Moreover, as a feature-wise loss, the negative structural similarity loss (SSIM) (Wang et al., 2004) can effectively match the luminance, contrast, and structure between two images. Hence, we combine the L1 loss and the negative SSIM as reconstruct loss to train our network, which can be expressed as Eqaution 4:


(4) $$\begin{array}{l} L_{rec}=\overset{3}{\underset{i=1}{\sum }}\left({||GT-J_{i}||}_{1}-SSIM\left(GT,J_{i}\right)\right) \end{array}$$

where *L*_*rec*_ represents the reconstruct loss and *GT* represents the ground truths. As shown in Figure 4, *J*_1_, *J*_2_, and *J*_3_ represents the final dehazed images *Out*, intermediate model-based dehazed images *J*_mod_, and the model-free dehazed images *J*_*free*_, respectively.

#### Distillation loss

In our PMGSDN, the dehazed images obtained from deeper layers play a role of teacher and transfer the knowledge to the shallow early-exit branches (performs as a student) within the network. Hence, the Distillation loss *L*_*dist*_ can be expressed as Eqaution 5:


(5) $$\begin{array}{l} L_{dist}={\left\|Out-J_{free}\right\|}_{1}+{\left\|Out-J_{\;\mathrm{mod}\;}\right\|}_{1}\\ \;+{\left\|J_{free}-J_{\;\mathrm{mod}\;}\right\|}_{1} \end{array}$$

where ||·||_1_ represents the L1 loss.

### Training and inference

During the training, the deeper AGFEBs are regarded as the teacher and they are utilized to guide the training of shallow AGFEB (student) by a distillation loss among the final dehazed images *Out*, intermediate model-based dehazed images *J*_mod_, and the model-free dehazed images *J*_*free*_. After the training, the whole PMGSND is optimized by model-based methods and model-free methods, which makes the PMGSDN to combine their merits. During the inference process, all of the early-exit branches are dropped so they do not bring additional parameters and computation.

Moreover, to make our manuscript readable, we list out the training process of the proposed algorithm and add it to the manuscript as a pseudocode (Table 2).

**Table 2:** The proposed algorithm.

**Training:**	
**Input:**	Hazy input image ***I***_*****in*****_, Corresponding haze-free image (Ground Truth, ***GT***), PMGSDN
**Output:**	The trained PMGSDN
Step 1	Start the training
Step 2	***I***_*****in*****_, → PMGSDN get ***A***_**0**_, ***t***_**0**_, and, ***J***_*****DCP*****_
Step 3	***A***_**0**_, ***t***_**0**_, and, ***J***_*****DCP*****_ → atmospheric scattering model, get ***I***_*****DCP*****_
Step 4	***I***_*****DCP*****_ → PMGSDN, get ***A***_**1**_, ***t***_**1**_, ***J***_*****free*****_, and, ***Out***
Step 5	***A***_**1**_, ***t***_**1**_, and, ***I***_*****DCP*****_ → atmospheric scattering model, get ***J***_*****mod*****_
Step 6	***GT***, ***Out***, ***J***_*****mod*****_, and, ***J***_*****free*****_ → Equation (4), get ***L***_*****rec*****_
Step 7	***Out***, ***J***_*****mod*****_, and, ***J***_*****free*****_ → Equation (5), get ***L***_*****dist*****_
Step 8	***L***_*****rec*****_ and ***L***_*****dist*****_ → Equation (6), get ***L***_*****loss*****_
Step 9	Back Propagation and update the PMGSDN
Step 10	Repeat the above steps until the end of the training
**Inference:**	
**Input:**	Hazy input image ***I***_*****in*****_, The trained PMGSDN
**Output:**	The final output ***Out***

## Experiments

To verify the effectiveness of the proposed PMGSDN, we quantitatively and qualitatively compare it with existing state-of-the-art methods, including DCP (He et al., 2011), DCPDN (Zhang and Patel, 2018), PSD (Chen et al., 2021), MSBDN (Dong et al., 2020), RefineD (Zhao et al., 2021), FFA (Qin et al., 2020), and DA (Shao et al., 2020). Moreover, we conduct an ablation study to verify the effectiveness of each part in PMGSDN.

### Datasets

In this article, we adopt the Indoor Training Set (ITS) in RESIDE (Li B. et al., 2019) to train our network, which contains 13990 synthetic hazy images and the corresponding clear images. During the training of the network, we adopt the Synthetic Objective Testing Set (SOTS) indoor datasets as the validation set, which contains 500 synthetic hazy images and the corresponding clear images. For testing, we use three synthetic datasets [I-HAZE (Ancuti C. et al., 2018), O-HAZE (Ancuti C. O. et al., 2018), and HazeRD (Zhang et al., 2017)] to evaluate the performance of the PMGSDN. Among them, the I-HAZE and O-HAZE contain 35 pairs of indoor and 45 pairs of outdoor hazy images. The HazeRD includes 75 pairs of hazy images degraded by different levels of haze. Considering the discrepancy that exists between synthetic and real-world hazy images, we further adopt real-world images from paper (Fattal, 2014) and Unannotated Real Hazy Images (URHI) (Shao et al., 2020) to evaluate the dehazing effect in real scenes.

### Implementation details

The proposed method is trained and tested in the Pytorch framework on a PC with the NIVIDIA GeForce RTX 3080 Ti. During the training, we resize input images to 256 × 256, set the batch size to 4, and train the network for 30 epochs. To effectively train the PMGSDN, we adopt the Adam optimizer with a default value for the attenuation coefficient to accelerate the training process (β_**1**_ = 0.9, β_**2**_ = 0.999). Moreover, we set the initial learning rate to 0.001 and reduce it by half every five epochs.

### Comparisons with state-of-the-art methods

#### Results on synthetic datasets

**Figure 5:**
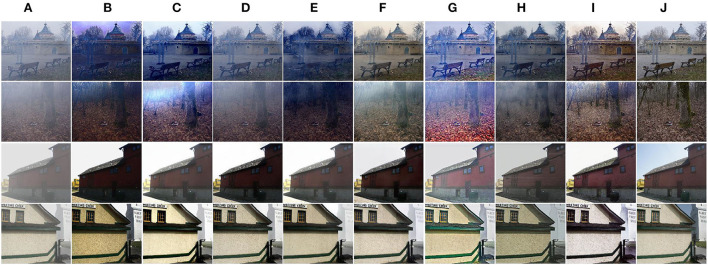
Qualitative comparisons on synthetic images from O-HAZE and HazeRD. The above two rows are images in O-HAZE and the others are images in HazeRD. **(A)** Haze. **(B)** DCP. **(C)** DCPDN. **(D)** MSBDN. **(E)** FFA. **(F)** DA. **(G)** PSD. **(H)** RefineD. **(I)** Ours. **(J)** GT.

Compared with indoor hazy images, outdoor hazy images have different scene depths and transmission maps. Hence, we pay more attention to the comparison results of outdoor images since the proposed PMGSDN is trained on indoor images. As shown in Figure 5, DCP effectively dehaze images but darken the results. Another model-based DCPDN estimates the transmission map and atmospheric light by CNN and generates better dehazed images but suffers from illumination distortion. By contrast, the model-free MSBDN restores dehazed images with better color fidelity but leads to a large amount of residual haze due to the over-fitting on training datasets. The FFA constructs a feature fusion attention network to effectively dehaze images but dims the brightness of results. Another method PSD can generate high contrast images but tend to overenhance the results due to simply guiding the pretrained network by priors. Compared with the above methods, the DA can restore dehazed images with satisfactory visual effect due to the use of domain adaption, and the RefineD restores dehazed images with vivid color but causes residual haze. Only our PMGSDN (see Figure 5I) acquires dehazed images with distinctive textures and abundant details, which verify the effectiveness of our method.

To further validate the performance of the proposed method, two metrics [peak signal-to-noise ratio (PSNR) and structural similarity (SSIM)] are adopted for quantitative comparison. As shown in Table 3, for I-HAZE, the DCP, DCPDN, and PSD perform poorly, which means that the abnormal illuminance and unwanted artifacts degrade the quality of dehazed images. By contrast, the end-to-end MSBDN and DA acquire a high value of PSNR and SSIM. Compared with other methods, the proposed PMGSDN achieves the highest value of these two metrics being 17.41 dB and 0.813, respectively. For O-HAZE, compared with the second-best method DA, the proposed PMGSDN improves the PSNR from 18.37 dB to 18.48 dB and improves the SSIM from 0.712 to 0.802, which validates its generalization ability. For HazeRD, the proposed PMGSDN achieves the PSNR and SSIM being 16.94dB and 0.867, which are slightly lower than that of RefineD.

**Table 3:** Qualitative comparisons on I-HAZE, O-HAZE, and HazeRD.

**Datasets**	**Metric**	**DCP**	**DCPDN**	**MSBDN**	**FFA**	**DA**	**PSD**	**RefineD**	**Ours**
I-Haze	PSNR	12.31 dB	14.27 dB	16.73 dB	13.10 dB	17.10 dB	12.92 dB	16.02 dB	17.41 dB
	SSIM	0.676	0.826	0.798	0.657	0.807	0.712	0.777	0.813
O-Haze	PSNR	14.94 dB	13.79 dB	18.08 dB	14.66 dB	18.37 dB	14.46 dB	17.71 dB	18.48 dB
	SSI	0.672	0.726	0.765	0.713	0.712	0.677	0.692	0.802
HazeRD	PSNR	13.26 dB	15.76 dB	15.23 dB	15.24 dB	16.88 dB	13.56 dB	17.81 dB	16.94 dB
	SSIM	0.795	0.781	0.839	0.745	0.818	0.742	0.850	0.867

Number in red and blue indicate the best and second-best results, respectively.

#### Results on real-world datasets

**Figure 6:**
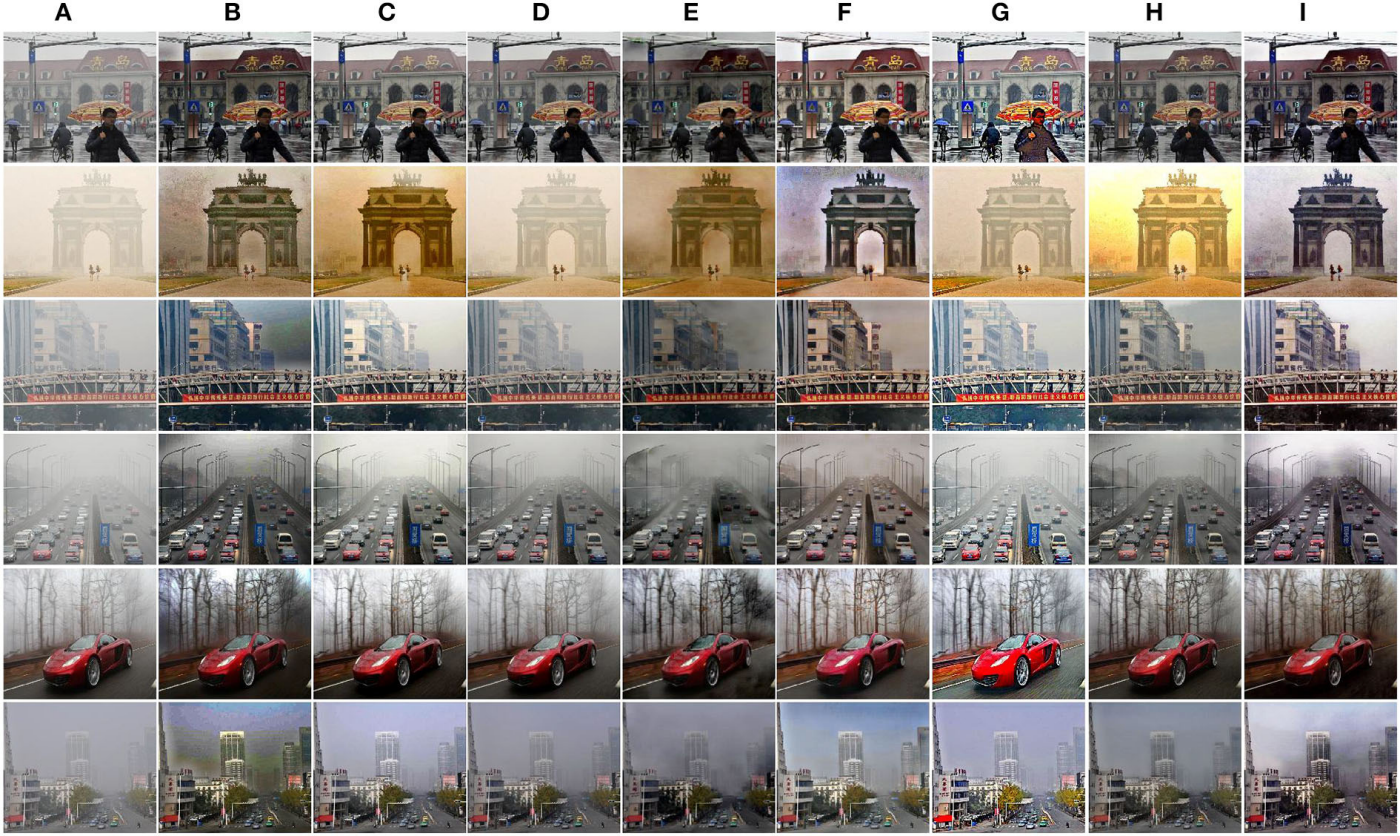
Qualitative comparisons on real-world images in URHI. **(A)** Hazy. **(B)** DCP. **(C)** DCPDN. **(D)** MSBDN. **(E)** FFA. **(F)** DA. **(G)** PSD. **(H)** RefineD. **(I)** Ours.

Considering the discrepancy between synthetic and real-world hazy images, we further validate the performance of our method on real-world images in Unannotated Real Hazy Images (URHI). As shown in Figure 6, DCP can produce dehazed images with distinct textures but inevitably causes halos and color distortions, which degrade the visual effect of results. Another model-based method DCPDN improves the brightness and contrast of dehazed images but simultaneously introduces some color changes since the atmospheric scattering model is a simplified model. By contrast, the model-free methods can restore dehazed images with better color fidelity but fail to deal with dense haze due to the lacking of extra knowledge as guidance. For example, MSBDN cannot effectively dehaze images due to over-fitting in synthetic datasets. Due to the feature fusion mechanism, FFA can effectively remove the haze in the local area of the image. However, due to the insufficient generalization ability of this method, it still causes residual haze and color changes in some regions. By building a bidirectional network to reduce the gap between synthetic and real-world hazy images, DA dehazes most haze and restores high quality results. Unfortunately, the sky regions are still degraded. Moreover, PSD simply guides the pretrained network by using multi priors, and the results are degraded by a large amount of residual haze. Another method RefineD embeds the DCP into the network and restores high quality images. Better than the above methods, the proposed PMGSDN (see Figure 6I) acquires dehazed images with distinctive textures and vivid color, which verify that it sufficiently exploits the features from different depths by self-distillation and combines the merits of model-based and model-free methods.

**Figure 7:**
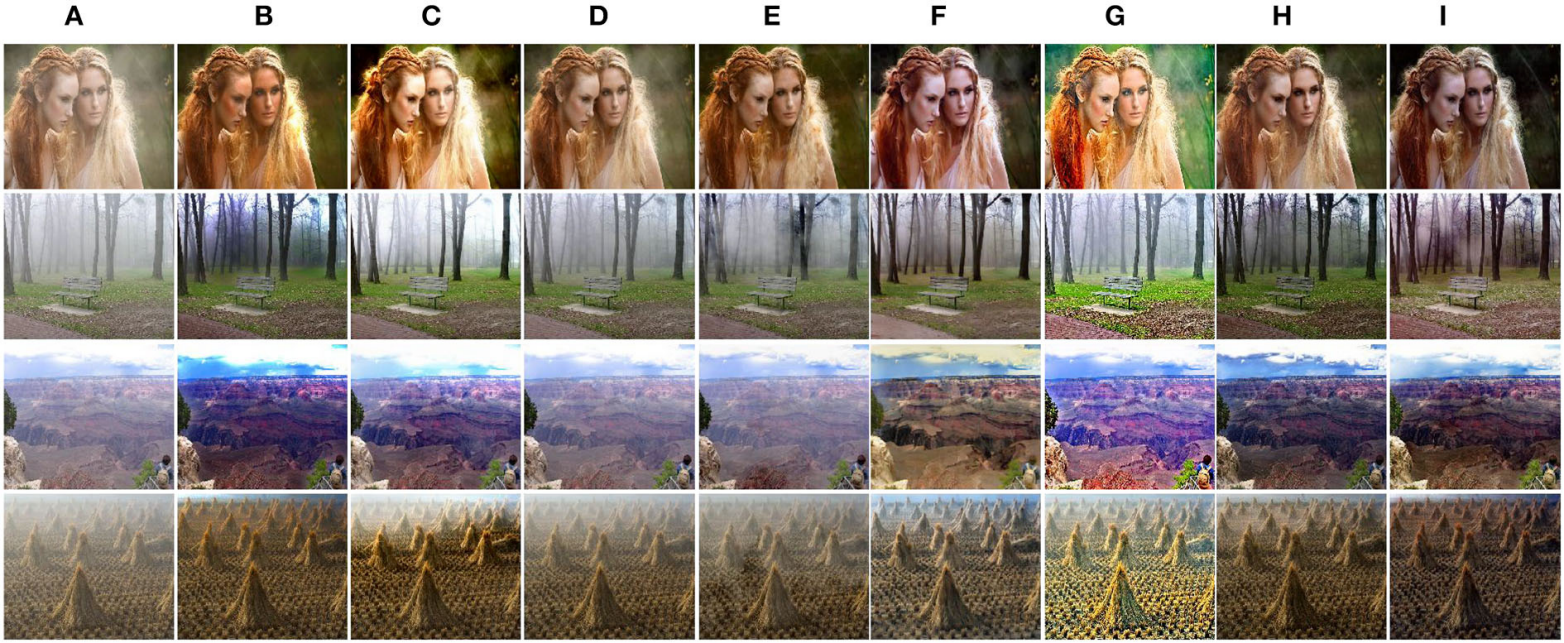
Qualitative comparisons on real-world images from Fattal (2014). **(A)** Hazy. **(B)** DCP. **(C)** DCPDN. **(D)** MSBDN. **(E)** FFA. **(F)** DA. **(G)** PSD. **(H)** RefineD. **(I)** Ours.

To further validate the generalization ability of our PMGSDN, we compare these methods on real-world images (Fattal, 2014). As shown in Figure 7, the DCP still effectively restores the textures but causes obvious color distortion in some regions. Another model-based DCPDN dehazes most haze but suffers from illumination oversaturation. By contrast, MSBDN cannot dehaze effectively in the real scene due to the lacking of knowledge guiding. Another model-free method FFA restores dehazed images with good color fidelity. However, this method neglects the generalization ability in the training process, which leads to the insufficient ability of the model. By contrast, DA removes most haze but suffers from slight color distortion. PSD suffers from illumination oversaturation and the sky regions contain some residual haze. Another method RefineD dehazes effectively and restores visually pleasing dehazed images. Better than the above methods, the proposed PMGSDN acquires high quality images with natural color and discriminative textures, which further shows that it conducts better generalization in real scenes.

In order to objectively evaluate the performance of the algorithm on real world datasets, we further select non-reference criteria that are widely used in image quality assessment for quantitative comparison. These criteria are Natural Image Quality Evaluator (NIQE) and Blind/Referenceless Image Spatial Quality Evaluator (BRISQUE), which can be used to evaluate the effect of haze, color shifts, illumination changes, and other image degraded phenomena. Table 4 gives the quantitative comparison results on the real-world images from paper (Fattal, 2014) and URHI datasets. For images in paper (Fattal, 2014), the proposed method achieves the best values of NIQE (Mittal et al., 2013) and BRISQUE (Mittal et al., 2012) being 2.891 and 13.56, respectively. For URHI datasets, the proposed method also achieves good dehazing results, with NIQE and BRISQUE of 3.705 and 21.38, respectively.

**Table 4:** Quantitative comparison results on the images in paper (Fattal, 2014) and URHI datasets.

**Datasets**	**Metric**	**Haze**	**DCP**	**DCPDN**	**MSBDN**	**FFA**	**DA**	**PSD**	**RefineD**	**Ours**
Images in paeper (Fattal, 2014)	NIQE	3.783	3.521	4.201	4.003	3.671	4.499	3.835	3.047	2.891
	BRISQUE	18.96	13.74	18.97	15.36	16.88	14.47	16.59	14.70	13.56
URHI	NIQE	4.715	3.982	4.058	4.605	3.707	4.388	3.822	3.511	3.705
	BRISQUE	33.73	27.62	27.89	27.36	27.53	21.79	24.26	22.64	21.38

The numbers in red, blue indicate the first and second-best results, respectively. Lower values of NIQE and BRISQUE represent better performance.

## Discussion

To verify the effectiveness of each part of the proposed PMGSDN, we conduct ablation studies to evaluate the performance of the following four key modules: the AGFEB, the guidance of preliminary dehazed images *J*_*DCP*_ generated by DCP, the guidance of intermediate dehazed images *J*_mod_ generated in a model-based way, and the guidance of intermediate dehazed images *J*_*free*_ generated in a model-free way. Hence, we construct the following variants: Variant A, the proposed method without the AGFEB, Variant B, the proposed method without the guidance of *J*_*DCP*_, Variant C, the proposed method without the guidance of *J*_mod_, Variant D, the proposed method without the guidance of *J*_*free*_, and Variant E, the proposed PMGSDN. We train these variants on ITS for 30 epochs and test them on I-HAZE and O-HAZE to evaluate the performance of each variant. As shown in Table 5, the proposed method achieves superior performance with PSNR and SSIM both on I-HAZE and O-HAZE, which validates that each part contributes to the PMGSDN.

**Table 5:** Results of ablation study.

	**Variant A**	**Variant B**	**Variant C**	**Variant D**	**Variant E**
IHAZE	15.85 dB	16.05 dB	16.72 dB	17.27 dB	17.41 dB
	0.728	0.719	0.738	0.759	0.813
OHAZE	16.24 dB	16.51 dB	16.33 dB	17.09 dB	18.48 dB
	0.702	0.647	0.692	0.697	0.802

## Conclusion

In this article, we propose a physical-model guided self-distillation network for single image dehazing named PMGSDN. First, we extract abundant features by the deep feature extraction network and acquire two intermediate dehazed images based on the model-based methods and model-free methods, respectively. Second, we embed the dark channel prior information to the network to combine the merits of both model-based methods and model-free methods to improve the dehazing effect. Finally, we adopt self-distillation strategy to improve the dehazing effect. For I-HAZE and O-HAZE datasets, the proposed method achieves the highest values of PSNR and SSIM being 17.41dB, 0.813, 18.48dB, and 0.802, respectively. For real-world images in URHI datasets, the proposed method also achieves the best value of BRISQUE being 21.38. The experimental results on both synthetic and real-world images show that the proposed PMGSDN dehazes more effectively and causes less distortions when compared with the state-of-the-art methods.

## Data availability statement

The raw data supporting the conclusions of this article will be made available by the authors, without undue reservation.

## Author contributions

All authors listed have made a substantial, direct, and intellectual contribution to the work and approved it for publication.

## Funding

This research was funded by the National Natural Science Foundation of China, grant number 61501470.

## Conflict of interest

The authors declare that the research was conducted in the absence of any commercial or financial relationships that could be construed as a potential conflict of interest.

## Publisher's note

All claims expressed in this article are solely those of the authors and do not necessarily represent those of their affiliated organizations, or those of the publisher, the editors and the reviewers. Any product that may be evaluated in this article, or claim that may be made by its manufacturer, is not guaranteed or endorsed by the publisher.
